# The APC/C Activator Cdh1p Plays a Role in Mitochondrial Metabolic Remodelling in Yeast

**DOI:** 10.3390/ijms24044111

**Published:** 2023-02-18

**Authors:** Ana Cláudia Leite, Maria Barbedo, Vítor Costa, Clara Pereira

**Affiliations:** 1 i3S—Instituto de Investigação e Inovação em Saúde, Universidade do Porto, Rua Alfredo Allen, 208, 4200-135 Porto, Portugal; 2 IBMC—Instituto de Biologia Celular e Molecular, Universidade do Porto, Rua Alfredo Allen, 208, 4200-135 Porto, Portugal; 3 ICBAS—Instituto de Ciências Biomédicas Abel Salazar, Universidade do Porto, Rua Jorge de Viterbo Ferreira 228, 4050-313 Porto, Portugal

**Keywords:** mitochondria, Cdh1p, proteomics, Yap1p, yeast

## Abstract

Cdh1p is one of the two substrate adaptor proteins of the anaphase promoting complex/cyclosome (APC/C), a ubiquitin ligase that regulates proteolysis during cell cycle. In this work, using a proteomic approach, we found 135 mitochondrial proteins whose abundance was significantly altered in the *cdh1*Δ mutant, with 43 up-regulated proteins and 92 down-regulated proteins. The group of significantly up-regulated proteins included subunits of the mitochondrial respiratory chain, enzymes from the tricarboxylic acid cycle and regulators of mitochondrial organization, suggesting a metabolic remodelling towards an increase in mitochondrial respiration. In accordance, mitochondrial oxygen consumption and Cytochrome *c* oxidase activity increased in Cdh1p-deficient cells. These effects seem to be mediated by the transcriptional activator Yap1p, a major regulator of the yeast oxidative stress response. *YAP1* deletion suppressed the increased Cyc1p levels and mitochondrial respiration in *cdh1*Δ cells. In agreement, Yap1p is transcriptionally more active in *cdh1*Δ cells and responsible for the higher oxidative stress tolerance of *cdh1*Δ mutant cells. Overall, our results unveil a new role for APC/C-Cdh1p in the regulation of the mitochondrial metabolic remodelling through Yap1p activity.

## 1. Introduction

Mitochondria are essential organelles that play a critical role in several cellular functions including ATP synthesis by the oxidative phosphorylation system (OXPHOS). The biogenesis of the OXPHOS system requires the concerted expression of the nuclear and the mitochondrial genomes [1]. In yeast, the mitochondrial proteome is largely dependent on substrate availability. The presence of glucose induces the catabolite repression of mitochondrial function [1,2]. The transition from fermentative to respiratory metabolism (known as diauxic shift) and shift to nonfermentable carbon sources trigger a major metabolic reorganization with the transcriptional up-regulation of many genes required to promote not only an increase in mitochondrial biogenesis and mitochondrial mass, but also a remodelling of mitochondria function towards a more respiratory mode, with an increase in OXPHOS complexes and tricarboxylic acid (TCA) enzymes [3,4,5]. The mitochondrial regulation is achieved mainly at the transcriptional level by the concerted regulation of multiple transcription factors by glucose-sensing signaling pathways (reviewed in [6]). Signaling pathways also seem to impact on mitochondrial metabolic reprogramming independently of the carbon source. By modulating the transcription of nuclear-encoded mitochondrial proteins, the cAMP-dependent protein kinase A (PKA) pathway regulates the mitochondrial enzyme content, and not the total mass, increasing the oxidative phosphorylation capacity of the cells [7,8]. Likewise, reduced TOR signaling increases mitochondrial oxygen consumption, in part, by up-regulating the translation of mitochondrial genome-encoded OXPHOS subunits, enhancing the density of OXPHOS complexes [9,10]. The type 2A-related serine-threonine phosphatase Sit4p is one of the TOR complex 1 (TORC1) downstream effectors that plays a role in mitochondrial glucose repression [11] and impacts on OXPHOS activity. Sit4p modulates the phosphorylation status of several mitochondrial proteins, including the ATP synthase catalytic beta subunit (Atp2p in yeast) [12]. In the absence of Sit4p, the phosphorylation of Atp2p leads to an increase in the ATP synthase levels, impacting the activity of the respiratory chain complexes and enhancing overall mitochondrial respiration [12].

We recently reported that the Atp2p levels increase in the absence of the anaphase-promoting complex/cyclosome (APC/C) activator Cdh1p [13]. APC/C is an E3 ubiquitin ligase responsible for the ubiquitin-dependent degradation of many cell cycle regulators [14,15], and its activity is primarily regulated through the temporal activation of two cofactors, Cdc20p and Cdh1p (also known as Hct1p) [16,17]. Cdc20p and Cdh1p carry conserved receptor domains to recognize specific sequence signals such as the destruction box and the KEN box that provide substrate selectivity [18]. Unlike Cdc20p, Cdh1p is not essential in yeast, though *cdh1*Δ cells exhibit a prolonged cell cycle and are sensitive to different types of stress, such as caffeine, alkalinity and hyperosmotic stress [19]. In addition to targeting mitotic regulators, emerging evidence suggests that Cdh1p has cell cycle-independent functions both in yeast [20] and mammals [21]. Although Atp2p is not an APC/C-Cdh1p direct target [13], the fact that its protein abundance is affected in cells lacking Cdh1p raises the question of whether Cdh1p may play a role in the regulation of mitochondrial function.

In the current study we performed a mitochondrial proteomic analysis and found that deletion of *CDH1* impacts on the abundance of many mitochondrial proteins in yeast. Overall, absence of Cdh1p promotes a shift towards a higher mitochondrial respiratory metabolism, which is dependent on the basic leucine zipper (bZIP) transcription factor Yap1p.

## 2. Results

### 2.1. CDH1 Deletion Leads to a Remodelling of the Mitochondrial Proteome and Promotes Mitochondrial Respiration

To evaluate the impact of APC/C-Cdh1p activity on yeast mitochondria, the mitochondrial proteome of wild type (wt) and *CDH1*-deleted cells was analysed by high-resolution mass spectrometry (HPLC-MS/MS). Quantification of mitochondrial proteins was performed with normalization based on total peptide amount. Cells were grown to mid-log phase under semi-respiratory conditions using galactose as a carbon source to obtain a higher mitochondrial mass, and mitochondria were then isolated by differential centrifugation. The proteomic data obtained gave a high level of replicate reproducibility with a total of 922 proteins previously reported as mitochondrial, representing a coverage of 90–100% depending on the reference proteome used [22,23]. Only these proteins were used for further data treatment.

**Figure 1:**
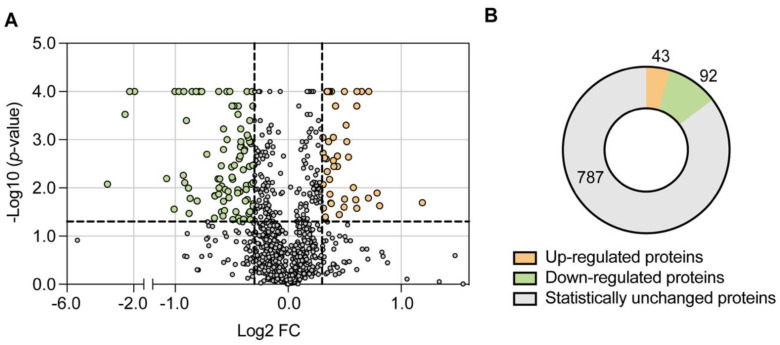
A total of 135 mitochondrial proteins exhibited significantly altered abundance in Cdh1p-deficient cells. (**A**) Volcano plot showing differentially expressed proteins in *cdh1*∆ mutant. Log-transformed *p*-values (*t*-test) are plotted against log-transformed fold change (FC). The horizontal dashed line marks a *p*-value of 0.05. Vertical dashed lines indicate a Log2 FC of ± 0.3. The up-regulated and down-regulated mitochondrial proteins are highlighted in orange and green, respectively. The plot was cropped between -1.4 and -1.2 Log2 FC to improve data visualization. (**B**) Diagram depicting the number of mitochondrial proteins up- and down-regulated in *cdh1*∆ mutant cells.

To evaluate overall changes in the mitochondrial proteome upon *CDH1* deletion, we used biological triplicate proteomic data for significance testing of the protein abundance changes in a pairwise manner. Student’s *t* test was used to identify differential protein expression between wt and *cdh1*Δ cells and represented in a volcano plot (Figure 1A). To analyse changes in protein abundance, a cut-off of *p*-value < 0.05 and an absolute log2 fold change (log2 FC) > 0.3 were applied. A total of 135 proteins exhibited altered protein abundance in the absence of Cdh1p activity, with 43 up-regulated and 92 down-regulated proteins (Figure 1B). Dataset S1 list the top up- and down-regulated mitochondrial proteins.

The proteins that increased the most in *cdh1*Δ cells are four succinate dehydrogenase subunits (Complex II), Sdh1p, Sdh3p, Sdh4p and Sdh6p. Among the most abundant proteins are also two Cytochrome *c* oxidase (Complex IV; Cox2p, Cox5p) subunits, two subunits of Cytochrome *bc*_1_ complex (Complex III; Qcr2p and Rip1p) as well as the NADH:ubiquinone oxidoreductase (equivalent to mammalian Complex I; Ndi1p). This shows that deletion of *CDH1* increases the abundance of proteins from all respiratory chain complexes. Among the most overrepresented proteins are also the respiratory chain soluble carrier Cytochrome *c* isoform 1 (Cyc1p), Aconitase (Aco1p) and the ADP/ATP translocator isoform (Aac1p), also involved in the respiratory metabolism. As we previously found, Atp2p was statistically significantly up-regulated in *cdh1*Δ cells, but stayed below our defined threshold.

**Figure 2:**
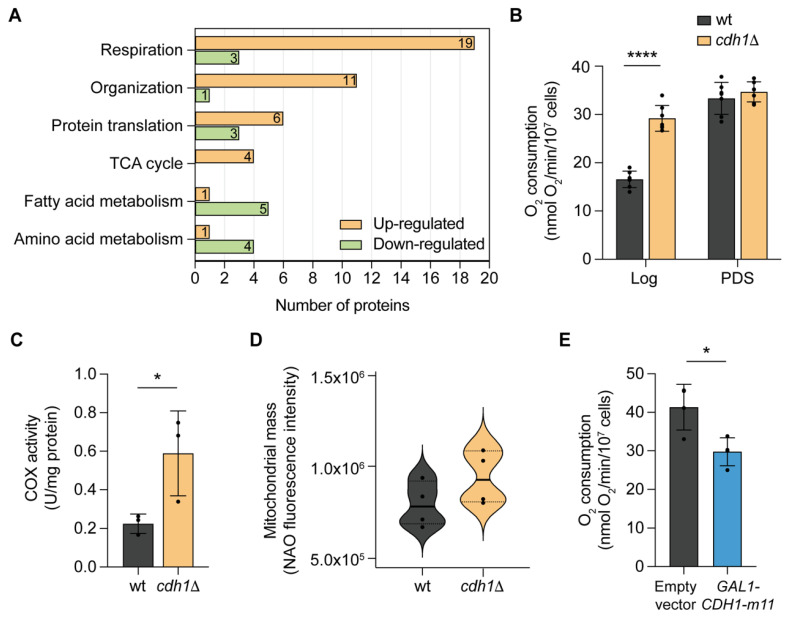
*CDH1* deletion promotes a metabolic remodeling towards an increased respiratory metabolism. (**A**) Gene ontology (GO)-term enrichment analysis on biological processes for statistically altered proteins using STRING v11.0. (**B**) Cells were grown until mid-log or post-diauxic shift (PDS) phase and the respiratory rate was obtained measuring oxygen consumption rate in whole cells. Values are the mean ± SD (*n* = 7); ****, *p* < 0.0001; *t*-test. (**C**) Cells were grown until mid-log phase and cytochrome *c* oxidase activity was determined by following the rate of cytochrome *c* oxidation. Values are the mean ± SD (*n* = 3); *, *p* < 0.05; *t*-test. (**D**) Cells were grown until early-log phase and the mitochondrial mass was determined by staining the cells with the dye nonyl acridine orange (NAO) and analyzed by flow cytometry (*n* = 4). (**E**) For the Cdh1p overexpression assay, cells were grown in raffinose media until mid-log phase and oxygen consumption rate measured after 3h of addition of 4% galactose. Values are the mean ± SD (*n* = 4); *, *p* < 0.05; *t*-test.

To identify the biological processes most impacted in *cdh1*Δ cells, a gene ontology (GO)-term enrichment analysis on biological processes was run using STRING v11.0 database [24]. This analysis showed that among the 43 up-regulated mitochondrial proteins the TCA cycle and mitochondrial respiration were the most represented processes (Figure 2A). Our results are consistent with the mitochondrial proteome analysis of yeast grown in respiratory conditions (versus fermentative) in which an overrepresentation of proteins associated to these processes have been reported [3,5,25]. We also found that proteins associated with mitochondrial protein synthesis and mitochondrial organization are enriched in the *cdh1*Δ mutant, namely several proteins involved in respiratory complexes assembly and two proteins involved in mitochondrial morphology, Fis1p and Dnm1p. Since both proteins are involved in fission, we analysed the mitochondrial network morphology in the *cdh1*Δ mutant but found no alterations in mitochondrial morphology (Figure S1). However, this was not entirely unexpected as increased Fis1p and Dnm1p abundance are also associated to the proteome remodelling that occurs upon the transition to a respiration metabolism, and this is not associated to mitochondrial fragmentation [3,25].

On the other hand, the 92 down-regulated proteins include proteins from diverse functional categories, with fatty acid metabolism (Cat2p and Oar1p among the most abundant in this category) and amino acid metabolism (glycine catabolism and aspartate synthesis) as the most relevant down-regulated biological process in the *cdh1*Δ mutant.

These results suggest that deletion of *CDH1* promotes a metabolic remodelling towards an increased respiratory metabolism, demonstrated by the up-regulation of proteins involved in energy generation. An increase in the abundance of proteins associated with the respiratory chain and TCA cycle are hallmarks in the transition from fermentative to respiratory growth conditions [3]. To confirm these results, oxygen consumption in the *cdh1*Δ mutant was evaluated in whole cells in the conditions used for the proteomic analysis. In accordance, the results showed a 1.8-fold increase in mitochondrial respiration in *cdh1*Δ cells compared to wt cells (Figure 2B). We also assessed the oxygen consumption rate of *cdh1*Δ cells from post-diauxic shift (PDS) cells when yeast switch their growth from fermentation to mitochondrial respiration. At PDS, the oxygen consumption rate in *cdh1*Δ cells was similar to that in wt cells (Figure 2B), indicating that Cdh1p does not regulate the normal derepression of respiratory genes at the diauxic shift. This suggests that either Cdh1p plays a role in mitochondrial function only in proliferating cells, or that it exhibits an early catabolite derepression.

The increase in mitochondrial respiration in mid-log *cdh1*Δ cells was further supported by the increased activity of the respiratory complex cytochrome *c* oxidase (COX) (from 0.22 U/mg protein in wt to 0.59 U/mg protein in *cdh1*Δ mutant; Figure 2C). Since the remodelling towards a more respiratory metabolism is often accompanied by an increase in mitochondrial biogenesis, we performed in vivo measurements of mitochondrial mass using nonyl acridine orange (NAO) fluorescence. We found that the mitochondrial mass is mildly increased in *cdh1*Δ cells when compared to wt cells, but the difference was not statistically significant (7.9 × 10^5^ ± 1.2 × 10^5^ to 9.4 × 10^5^ ± 1.5 × 10^5^, mean ± SD, Figure 2D). This indicates that the increased mitochondrial respiration in *cdh1*Δ cells is mostly due to an increase in the respiratory capacity of mitochondria than to an increase in mitochondrial mass. Furthermore, overexpression of a constitutively active Cdh1-m11 form (lacking the 11 Cdk inhibitory-phosphorylation sites) [26] results in a decrease in mitochondrial respiration compared to wt cells expressing the empty vector (Figure 2E).

### 2.2. The Transcription Factors Yap1p and Rpn4p Mediate the Induction of Mitochondrial Respiration in cdh1Δ Cells

**Figure 3:**
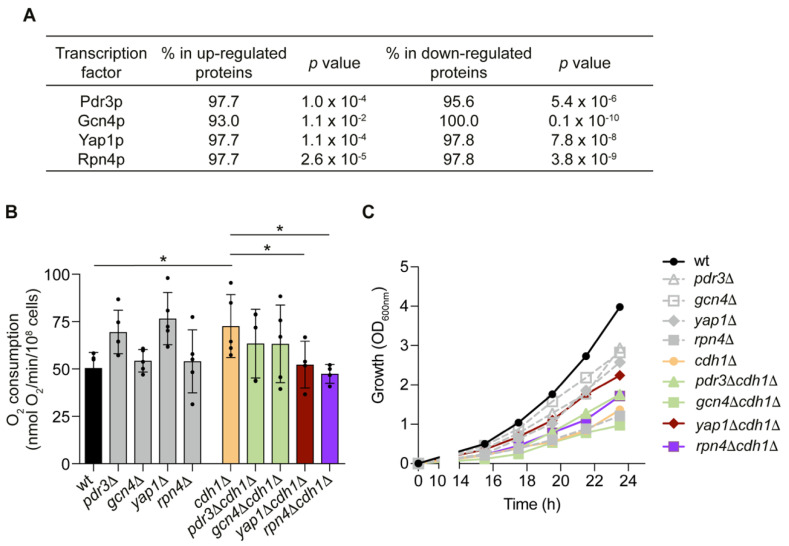
The transcription factors Yap1p and Rpn4p are required for the increase in mitochondrial respiration in the *cdh1*∆ mutant. (**A**) Table showing the YEASTRACT+ predicted transcription factors that might regulate the mitochondrial proteins altered in *cdh1*∆ cells. (**B**) Cells were grown until late-log phase and the respiratory rate was obtained measuring oxygen consumption rate in whole cells. Values are the mean ± SD (*n* = 4); *, *p* < 0.05; one-way ANOVA followed by Tukey’s multiple-comparison test. (**C**) The growth of the indicated strains was monitored over time by optical density (OD_600nm_) measurements. Values are the mean (*n* = 3).

Since Cdh1p is part of an E3 ubiquitin ligase complex, it is possible that part of the mitochondrial proteins could be regulated by Cdh1p-mediated proteolysis. However, given the high number of altered proteins, with a high proportion of down-regulated proteins, it is most likely that Cdh1p is impacting on mitochondrial proteins indirectly, possibly through the modulation of transcription factor(s). The repository YEASTRACT+ (Yeast Search for Transcriptionally Regulators And Consensus Tracking) [27] was used to predict the transcription factors that might be responsible for the protein expression patterns in *cdh1*Δ cells. This led to the identification of four transcription factors as possibly regulating the adaptive responses to *CDH1* deletion (Figure 3A). This list includes Pdr3p (regulator of the pleiotropic drug resistance), Gcn4p (regulator of amino acid biosynthetic genes in response to amino acid starvation), Yap1p (regulator of the oxidative stress response) and Rpn4p (regulator of the proteasome). To investigate whether these transcription factors mediate the effects of *CDH1* deletion on mitochondrial function, double mutant strains deleted both in *CDH1* and in the individual transcription factors were constructed. The absence of the selected transcription factors on the *cdh1*Δ mitochondrial phenotype was first evaluated by measuring oxygen consumption rate. Our results show that deletion of *PDR3* and *GCN4* did not significantly affect *cdh1*Δ high oxygen consumption. On the other hand, both *YAP1* and *RPN4* deletion restored *cdh1*Δ respiration to wt levels (Figure 3B). We also assessed the impact of the transcription factors deletion on cell growth by measuring optical density (OD) over time (Figure 3C). For quantitative evaluation of growth and statistical analysis purposes, the area under each growth curve was also calculated (AUC; values in Figure S2). Cells lacking Cdh1p exhibited a significant growth delay when compared to wt cells, which can be attributed to the accumulation of cell cycle progression substrates like Clb2p and Ase1p [16]. Deletion of *PDR3*, *RPN4* and *YAP1* improved the growth of *cdh1*Δ cells. This effect was more significant for *YAP1* deletion which, despite not reverting *cdh1*Δ growth to wt levels (AUC of 19.4), almost doubled the AUC from 6.7 in *cdh1*Δ cells to 12.5 in *yap1*Δ*cdh1*Δ cells. This suggests that *YAP1* genetically interacts with *CDH1* and contributes to the *cdh1*Δ mutant slow growth phenotype.

**Figure 4:**
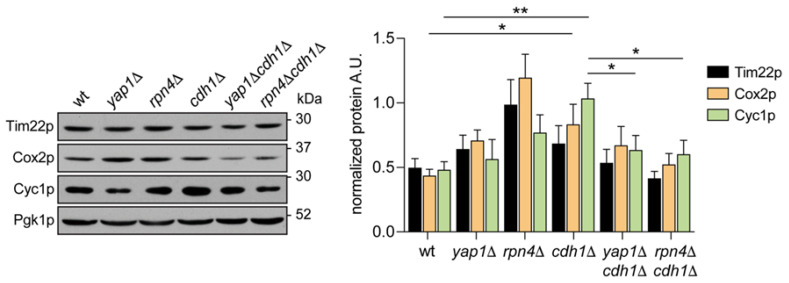
The up-regulation of Cyc1p in the *cdh1*∆ mutant is mediated by the transcription factors Yap1p and Rpn4p. Cells with the indicated genotypes were grown until mid-log phase and Cox2p and Cyc1p levels on total proteins extracts were assessed by immunodetection. Tim22p (inner mitochondrial protein unchanged in *cdh1∆* cells) is shown as control of mitochondrial mass. A representative blot is shown. Graph represents the relative amount of Tim22p, Cox2p and Cyc1p normalized to Pgk1p. Values are the mean ± SEM (*n* = 5); *, *p* < 0.05; **, *p* < 0.01; *t*-test.

To further evaluate this functional relationship, we assessed how the deletion of *RPN4* and *YAP1* affected the expression of mitochondrial proteins previously identified as up-regulated (Cyc1p and Cox2p) in *cdh1*Δ cells. Cyc1p and Cox2p were among the proteins identified by YEASTRACT+ as potentially transcriptionally regulated by Yap1p and Rpn4p. Tim22p, which was found unaltered *cdh1*Δ cells, was also analysed as a control of mitochondrial mass. Accordingly, we found Cyc1p and Cox2p, but not Tim22p, accumulated at higher levels in proliferating *cdh1*Δ cells (Figure 4). Our results also show that Cyc1p levels were significantly decreased after deletion of both *YAP1* and *RPN4* in *cdh1*Δ cells. Cox2p levels also decreased in the double mutants when compared with *cdh1*Δ cells, but the difference was not statistically significant (Figure 4).

Overall, these results suggest that transcription factors Yap1p and Rpn4p function as Cdh1p downstream effectors in the regulation of mitochondrial protein levels. Interestingly, Yap1p and Rpn4p are functionally related, as *YAP1* itself contains a Proteasome Associated Control Elements (PACE) sequence in its promotor targeted by Rpn4p [28], while *RPN4*, in turn, can be transcriptionally regulated by Yap1p [29].

### 2.3. CDH1 Deletion Does Not Impact on Rpn4p Activity

Yeast Rpn4p is a C_2_H_2_ zinc finger transcription factor that is responsible for the expression of genes associated with proteasome biogenesis and activity and with ubiquitin-dependent proteolysis [30,31]. Inhibition of proteasome activity results in a Rpn4p stabilization, which binds to PACE sequences found in Rpn4p-recognized promotors, up-regulating its target genes [30].

**Figure 5:**
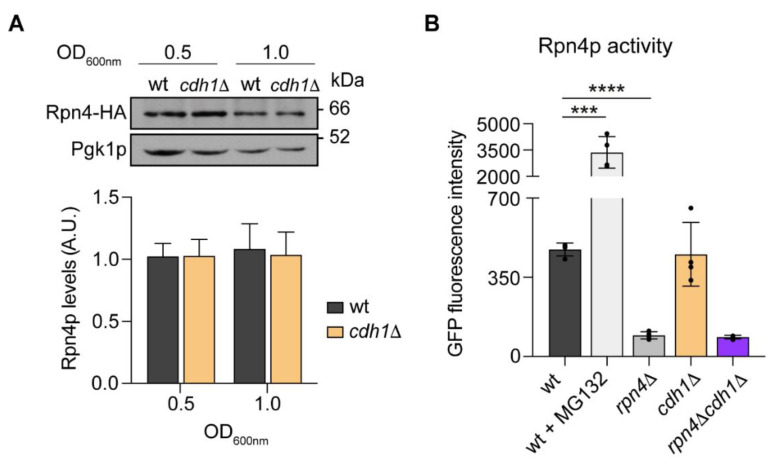
Rpn4p levels or activity are not altered in *cdh1*∆ mutant cells. (**A**) Wt or *cdh1*Δ cells endogenously expressing hemagglutinin (HA)-tagged Rpn4p were grown to early and mid-log phase and analyzed by immunoblotting using anti-HA and anti-Pgk1p (loading control) antibodies. A representative blot is shown. Graph represents the relative amount of Rpn4-HA normalized to Pgk1p. Values are the mean ± SEM (*n* = 4). (**B**) Cells with the indicated genotypes harboring a GFP reporter for Rpn4p activity were grown to early-log phase. As a positive control, wt cells were treated with the proteasome inhibitor MG132. The GFP fluorescence intensity was determined by flow cytometry. The values are the mean ± SD (*n* = 4), ***, *p* < 0.001; ****, *p* < 0.0001; *t*-test.

We next investigated the hypothesis that the direct targeting of Rpn4p by APC/C-Cdh1p may account for the mitochondrial alterations induced by *CDH1* deletion. For that, the levels of Rpn4p were evaluated by Western blot, in cells expressing HA-tagged endogenous Rpn4p. We found that Rpn4p stability was not increased by *CDH1* deletion (Figure 5A), suggesting that Rpn4p is not a direct substrate of APC/C-Cdh1p. In addition, it suggests that Rpn4p is not more active in *cdh1*Δ cells as Rpn4p stabilization is associated to its activity [32]. To confirm this, we assessed Rpn4p transcriptionally activity using a Rpn4p-driven GFP reporter [33]. As a positive control, wt cells were incubated with 60 μM of the proteasome inhibitor MG132 for 2 h. As shown in Figure 5B, GFP fluorescence was significantly elevated in MG132-treated cells while *RPN4*-deleted cells showed a strong decrease, validating the reporter specificity. However, loss of Cdh1p did not affect Rpn4p transcriptional activity, supporting the hypothesis that Rpn4p is not more active in *CDH1* deleted cells. These results suggest that Rpn4p activation is not the primary cause leading to Cdh1p-mediated up-regulation of mitochondrial respiration.

### 2.4. Yap1p Is More Active in cdh1∆ Cells

Yeast Yap1p is a leucine zipper (bZIP) transcription factor that activates the expression of genes encoding several antioxidant proteins [34]. Yap1p is activated in response to different reactive oxygen species (ROS), such as hydrogen peroxide (H_2_O_2_), by a mechanism that inhibits its nuclear export, thus promoting Yap1p nuclear accumulation and activation [35,36,37]. In addition to its well-known role in the oxidative stress response, Yap1p is also involved in the yeast response to metals and unrelated drugs [35] and seems to play a role in mitochondrial regulation [38,39,40].

**Figure 6:**
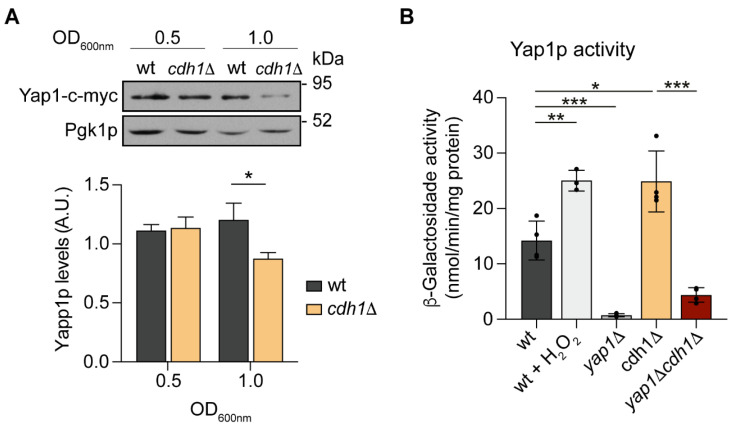
*CDH1* deletion leads to Yap1p activation. (**A**) yap1Δ and yap1Δcdh1Δ cells harboring pRS315-Yap1-c-Myc plasmid were grown to early- and mid-log phase and total protein extracts were separated by SDS-PAGE and analyzed by immunoblotting using anti-c-myc and anti-Pgk1p. A representative blot is shown. Graph represents the relative amount of Yap1-c-myc normalized to Pgk1p. Values are the mean ± SEM (*n* = 4); *, *p* < 0.05; *t*-test. (**B**) Cells with the indicated genotypes harboring pRS415-AP-1-CYC1-LacZ reporter were grown to mid-log phase. As a positive control, wt cells were exposed to hydrogen peroxide (H_2_O_2_). The specific β-galactosidase activity was determined using o-nitrophenyl-β-d-galactopyranoside (ONPG) as a substrate. The values are the mean ± SD (*n* = 4), *, *p* < 0.05; **, *p* < 0.01; ***, *p* < 0.001; *t*-test.

We next investigated the hypothesis that the direct targeting of Yap1p by APC/C-Cdh1p may account for the mitochondrial alterations induced by *CDH1* deletion. For that, we compared the steady-state level of the Yap1p protein in wt and *cdh1*Δ cells. The Yap1-9Myc protein was expressed from a vector under the regulation of its native promoter in the *yap1*Δ and *yap1*Δ*cdh1*Δ mutants. As shown in Figure 6A, no difference was detected in the protein levels of Yap1p at OD_600nm_ 0.5 suggesting Yap1p is not a Cdh1p direct substrate. In addition, the levels of Yap1p were also not affected by expression of a constitutively active Cdh1-m11 form (lacking the 11 Cdk inhibitory-phosphorylation sites) [26] or after mutation in a potential APC/C recognition motif predicted using GPS-ARM 1.0 (Figure S3). Intriguingly, at OD_600nm_ 1.0 Yap1p levels were even decreased in cells lacking Cdh1p (Figure 6A). It was previously reported that Yap1p activity is mostly controlled by the disruption of Yap1p nuclear export without affecting protein levels [35,41]. However, a decrease in Yap1p protein levels is often observed following its activation [35,41,42]. Therefore, our results suggest that Yap1p is not a direct Cdh1p target, but its transcriptional activity might be indirectly regulated by Cdh1p. To investigate this hypothesis we monitored Yap1p transcriptional activity in *cdh1*Δ cells, using a Yap1p-dependent lacZ reporter (pRS415-AP-1-CYC-LacZ) [41]. As a positive control, wt cells were treated with 5 mM H_2_O_2_ for 1.5h, which triggered a significant increase in β-Galactosidase activity (Figure 6B). On the other hand, the Yap1p-dependent β-Galactosidase activity in *yap1*Δ cells was dramatically decreased, confirming the reporter specificity. Notably, the results showed a 1.7-fold increase in β-Galactosidase activity in *cdh1*Δ cells compared to wt cells, indicating that Yap1p transcriptional activity is increased in cells lacking Cdh1p.

### 2.5. Yap1p Mediate the Oxidative Stress Resistance of cdh1∆ Cells

**Figure 7:**
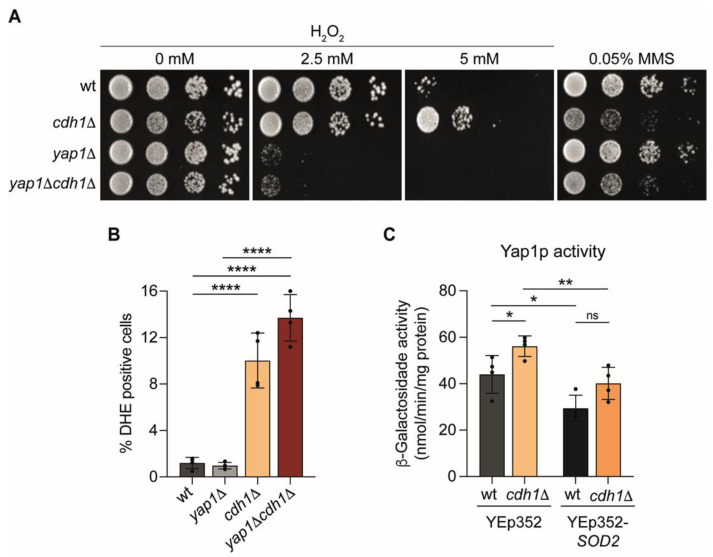
Yap1p mediates the oxidative stress resistance of the *cdh1Δ* strain. (**A**) H_2_O_2_ and methyl methanesulfonate (MMS) sensitivity of wt, *cdh1*Δ, *yap1*Δ and *yap1*Δ*cdh1*Δ cells. Cells were grown until mid-log phase and ten-fold dilutions were spotted onto YPGal plates without a stressor or with 2.5–5 mM H_2_O_2_ or 0.05% MMS. Plates were incubated at 26 °C for 2 days. A representative image is shown (*n* = 3). (**B**) Cells with the indicated genotypes were grown until early-log phase and cellular ROS levels were assessed by flow cytometry using the fluorescent probe dihydroethidium (DHE). The values are the mean ± SD (*n* = 4), ****, *p* < 0.0001; one-way ANOVA followed by Tukey’s multiple-comparison test. (**C**) Cells with the indicated genotypes harboring pRS415-AP-1-CYC1-LacZ reporter were grown to mid-log phase. The specific β-galactosidase activity was determined using o-nitrophenol-β-d-galactopyranoside (ONPG) as a substrate. The values are the mean ± SD (*n* = 4), ns, not significant, *, *p* < 0.05; **, *p* < 0.01; *t*-test.

Since Yap1p is a major oxidative stress response regulator in yeast, we asked whether its increased transcriptional activity in *cdh1*Δ cells led to an increase in oxidative stress resistance. To test this hypothesis cells were grown in solid media in the presence of H_2_O_2_. As expected, deletion of *YAP1* dramatically increases the H_2_O_2_ sensitivity (Figure 7A). In contrast, cells lacking Cdh1p presented a higher oxidative stress resistance compared to wt cells, particularly evident at the higher H_2_O_2_ concentration used. The increase in H_2_O_2_ resistance was dependent on Yap1p since its deletion in *cdh1*Δ cells restored oxidative stress sensitivity (Figure 7A). In contrast, *cdh1*Δ cells, but not *yap1*Δ cells, were more sensitive to methyl methanesulfonate (MMS) comparing to wt (Figure 7A), as reported for several other stressors [19]. These findings indicate that *cdh1*Δ cells are unexpectedly resistant to oxidative stress, and that this occurs due to Yap1p activation.

Under physiological conditions, mitochondria serve a major source of ROS that are mainly generated from the mitochondrial respiratory chain as a normal consequence of aerobic respiration [43,44]. Since *CDH1* deletion led to an up-regulation of mitochondrial respiration, we investigated its effect on ROS levels using dihydroethidium (DHE) as a probe that becomes fluorescent upon oxidation by superoxide radicals and hydrogen peroxide. At early-log phase, approximately 1% of wt cells exhibited ROS accumulation, whereas 10% of *cdh1*Δ cells displayed DHE staining (Figure 7B). ROS levels in the *cdh1*Δ*yap1*Δ double mutant were similar to those in *cdh1*Δ cells (Figure 7B). Since *YAP1* deletion lowered the increase in mitochondrial respiration in *cdh1*Δ cells, but not the ROS levels, we questioned whether the higher ROS levels in *cdh1*Δ cells may underlie Yap1p activation in these cells and precede the mitochondrial remodelling. To test this hypothesis, we analysed Yap1p transcriptional activity in *cdh1*Δ cells after overexpression of the mitochondrial superoxide dismutase (Sod2p) using Yep352-*SOD2* plasmid [45]. Overexpression of *SOD2* decreased the Yap1p-dependent β-Galactosidase activity in *cdh1*Δ cells comparing to cells expressing the empty-vector (Figure 7C). However, *SOD2* overexpression did not fully lower the Yap1p activity in the *cdh1*Δ mutant to the levels observed in wt cells overexpressing *SOD2*. This result led us to hypothesize that in *cdh1*Δ cells Yap1p is transcriptionally more active, leading to an increase in mitochondrial respiration, which results in higher mitochondrial ROS production. This in turn, in a positive feedback loop, further favours Yap1p activation in these cells.

## 3. Discussion

In this work we investigated the role of Cdh1p in the control of mitochondrial function using a proteomic approach. Cdh1p has a well-known role in ubiquitination of cell cycle substrates, regulating cell cycle processes such as G1/S transition and mitotic exit [17]. This study provides for the first time evidence that Cdh1p also plays a role in the regulation of mitochondrial functional remodelling and provides a global overview of the specific mitochondrial changes elicited by *CDH1* deletion. We found that deletion of *CDH1* causes a shift in mitochondrial proteome composition to promote a more respiratory mode, which was confirmed by measuring oxygen consumption and COX activity. Besides the canonical functions of APC/C, some studies in mammalian cells point to a role for Cdh1p in the regulation of metabolism and mitochondrial morphology [46,47]. APC/C-Cdh1 impacts on mitochondrial morphology by ubiquitinating Drp1 (the Dnm1p homologue), contributing to the maintenance of a dynamic balance between mitochondrial fission and fusion during mitotic exit [46]. Though we also found an increase in Dnm1p levels in cells lacking Cdh1p, due to the high number of mitochondrial proteins altered in the *cdh1*Δ mutant (135), with about two-thirds being down-regulated, it is more likely these are indirect effects. However, we cannot discard the hypothesis that among the up-regulated proteins some might be direct targets and subject to Cdh1p-regulated proteolysis. In fact, many mitochondrial proteins have potential Cdh1p canonical recognition motifs. However, the APC/C motifs are very common in the proteome [48] and, thus, are not strong substrate predictors and need to be experimentally validated.

The up-regulation of mitochondrial respiration in *cdh1*Δ mutant was suppressed upon deletion of genes encoding the transcription factors Yap1p or Rpn4p, supporting an indirect regulation of mitochondrial function. Though both Yap1p and Rpn4p, a downstream target of Yap1p [49], were required for mitochondrial functional remodelling in the *cdh1*Δ mutant, only Yap1p was found to be more active in these cells. Due to the functional relation between Yap1p and Rpn4p, Rpn4p may contribute to Yap1p effects, but the up-regulation of Yap1p function is likely the main trigger for the mitochondrial alterations in *cdh1*Δ cells. Yap1p is the main oxidative stress response regulator in yeast, but several works point for a potential role for Yap1p in mitochondrial function. Indeed, it was demonstrated that the transcription factor Yap1p is directly involved in the regulation of iron export from the mitochondria [38] and plays a role in the mitochondrion-to-nucleus signaling during growth on ethanol [39]. Importantly, Yap1p overexpression leads to an increase in the abundance of mitochondrial proteins associated to respiration [39], supporting our observations that increased Yap1p activity can lead to an enhancement in mitochondrial respiration in *cdh1*Δ cells. Interestingly, in the same study, authors also report Yap1p overexpression triggers alterations in proteins associated with cell cycle and growth regulation. Though Cdh1p can have cell cycle-independent functions, its main role is the regulation of cell cycle progression. Since we and others have found a synchronization between cell cycle progression and mitochondrial respiration in yeast [13,50], it will be interesting to assess if the role of Cdh1p in the regulation of mitochondrial function is cell cycle-independent or occurs during cell cycle progression. In fact, the oxygen consumption during cell cycle progression in lowest in G1, the phase in which Cdh1p is more active [13,50]. Likewise, the lower effect in the mitochondrial proteome remodelling in the *cdh1*Δ mutant compared to the transition to growth in respiratory substrates fits well with the maximum oscillations found in mitochondrial respiration during cell cycle progression (about 1.3 fold) [13]. In addition, Cdh1p does not seem to play a role in the traditional diauxic shift transition to respiration, as it did not affect the yeast respiration in PDS phase. In addition, Yap1p and Rpn4p are not important players in mitochondrial transcriptional regulation at this phase, with Msn2p and Msn4p [51], Cat8p [52] and Sip4p [53] as the main transcriptional factors involved in mitochondrial derepression. This suggests that Cdh1p impacts on mitochondrial respiration in proliferating cells independently of the canonical carbon source-responsive pathways.

A remaining question Is also how APC/C-Cdh1p regulates the activity of Yap1p to promote the induction of mitochondrial respiration. We provide evidence that *CDH1* deletion affects Yap1p activity, but not its protein levels. It is therefore possible that Cdh1p may regulated the proteins involved in the regulation of Yap1p activity/nuclear export. Since we found that *cdh1*Δ cells exhibit higher ROS levels than wt cells, it is also possible that Yap1p is being activated by the oxidative environment of *cdh1*Δ cells. Curiously, though Yap1p seem involved in the up-regulation of respiration in *cdh1*Δ cells, Yap1p transcriptional activity has been described to be also induced by mitochondrial respiration [54,55]. The mitochondria respiratory chain is the major source of endogenous ROS [44], and therefore transition to mitochondrial respiratory growth is accompanied by the induction of cellular antioxidant defences, which allows the cells to become intrinsically more tolerant to oxidants than fermenting grown yeast [54,55]. Activation of Yap1p in *cdh1*Δ cells may allow the coordination of mitochondrial respiration with oxidant resistance, particularly vital if the regulation of mitochondrial function by Cdh1p occurs during cell division, as ROS are particularly harmful to replicating DNA [56] and can lead to cell cycle arrest [57]. Interestingly, two additional transcription factors, Tos4p and Pdr3p, implicated in the DNA damage response were reported to be positively regulated by Cdh1p [58]. Together with our results, this suggests Cdh1p may play a broader role than believed in the cellular transcriptional responses to different environmental stresses.

In conclusion, our study reveals a novel role for Cdh1p in the regulation of mitochondrial metabolic remodelling contributing to our understanding of the signalling pathways controlling cellular energy homeostasis. Regulation of mitochondrial metabolism occurs after glucose exhaustion, in the presence of alternative respiratory carbon sources and even during cell cycle progression [13,50]. Mitochondrial metabolic remodelling also occurs in response to diverse signalling pathways [7,8,9,10,11] reinforcing the importance of fine-tuning mitochondrial function with energetic demands. We also report that Cdh1p impacts on Yap1p transcriptional activity, which underlies both the *cdh1*Δ mutant resistance to oxidative stress and the up-regulated mitochondrial respiration. The integration of mitochondrial function with the induction of antioxidant defences through Yap1p may be important to maintain the cellular redox balance in *cdh1*Δ cells.

## 4. Materials and Methods

### 4.1. Yeast Strains and Growth Conditions

The *Saccharomyces cerevisiae* strains used are all BY4741 derivative and are listed in Table S1. To generate *cdh1*∆::*HIS3* strain, *cdh1∆::KanMX4* was transformed with a DNA fragment containing *HIS3MX*. To construct double mutant strains, the DNA fragment containing *cdh1*∆::*HIS3* was amplified and transformed in the deletion strains. To generate Rpn4-HA*cdh1*Δ::*kan* strain, Rpn4-HA:*HIS3* was transformed with a DNA fragment containing *cdh1∆::KanMX4*. Strains were transformed by the standard lithium acetate procedure [59]. Gene deletion was confirmed by PCR. For overexpression of Cdh1-m11 and Sod2p, cells were transformed with the plasmids pRS416-GALL-3HA-Cdh1-m11 [26] and Yep352-SOD2 [45], respectively. 

Cells were grown in rich medium [YPGal: 2% (*w/v*) galactose, 1% (*w/v*) yeast extract, 2% (*w/v*) bactopeptone] or synthetic complete medium [SC: 0.67% (*w/v*) Bacto-yeast nitrogen base w/o amino acids, 2% (*w/v*) glucose and 0.2% (*w/v*) Dropout mix] lacking uracil/leucine, as appropriate. For Cdh1-m11 overexpression, cells were grown in YPRaff medium [2% (*w/v*) raffinose, 1% (*w/v*) yeast extract, 2% (*w/v*) bactopeptone] overnight until mid-log phase and cultured with 4% galactose for 3h before oxygen consumption analysis. Cultures were routinely grown at 26 °C in an orbital shaker at 140 r.p.m.

### 4.2. Mitochondrial Isolation

For isolation of an enriched mitochondrial fraction, wt and *cdh1*Δ cells were grown to mid-log phase (OD_600nm_ = 1.4) in YPGal medium and digested enzymatically with zymolyase (5 mg/g of cells) at 37 °C for 30 min. The homogenized spheroplasts were subjected to differential centrifugation basically as described in [60].

### 4.3. Protein Identification by HPLC-MS/MS

Biological triplicates from wt and *cdh1*Δ cells were solubilized with 100 mM Tris pH 8.5, 1% (*w/v*) sodium deoxycholate, 10 mM tris(2-carboxyethyl) phosphine (TCEP) and 40 mM chloroacetamide for 10 min at 95 °C at 1000 r.p.m. Each sample was processed for proteomics analysis following the solid-phase-enhanced sample-preparation (SP3) protocol as described in [61]. Enzymatic digestion was performed with Trypsin/LysC (2 μg) overnight. Protein identification and quantitation was performed by nanoLC-MS/MS composed by an Ultimate 3000 liquid chromatography system coupled to a Q-Exactive Hybrid Quadrupole-Orbitrap mass spectrometer (ThermoFisher Scientific, Waltham, MA, USA), as previously described [62]. This equipment is composed of an Ultimate 3000 liquid chromatography system coupled to a Q-Exactive Hybrid Quadrupole-Orbitrap mass spectrometer (ThermoFisher Scientific, Waltham, MA, USA).

The raw data were processed using Proteome Discoverer 2.5.0.400 software (ThermoFisher Scientific, Waltham, MA, USA) and searched against the UniProt database for the *Saccharomyces cerevisiae* Proteome 2020_03 together with a common contaminant database from MaxQuant (version 1.6.2.6, Max Planck Institute of Biochemistry, Martinsried, Germany). The Sequest HT search engine was used to identify tryptic peptides. Peptide confidence was set to high. The processing node Percolator was enabled with the following settings: maximum delta Cn 0.05; decoy database search target FDR 1%, validation based on q-value. Protein label free quantitation was performed with the Minora feature detector node at the processing step. Precursor ions quantification was performing at the processing step with the following parameters: Peptides to use unique plus razor, precursor abundance based on intensity and normalization based on total peptide amount.

### 4.4. Mitochondrial Mass Analysis

The total mitochondrial mass was determined using 10-N-Nonyl acridine orange (NAO, Invitrogen, Waltham, MA, USA), a dye that binds to cardiolipin present specifically on the mitochondrial membrane [63]. Briefly, wt and *cdh1*Δ cells were grown to mid-log phase in YPGal medium and incubated in culture medium containing 10 μM NAO for 30 min. Fluorescence intensity measured using the BD Accuri C6 flow cytometer. Data were analysed with FlowJo v10 software version.

### 4.5. Oxygen Consumption Rate and COX Activity

The oxygen consumption was measured polarographically in whole cells resuspended in PBS buffer, from cultures grown in YPGal medium to mid-log or PDS phase, using a Clark-type oxygen electrode coupled to an Oxygraph plus system (Hansatech, King’s Lynn, United Kingdom).

Data were analysed using the OxyTrace^+^ software. The respiratory rate was obtained by dividing the oxygen consumed per min by the number of cells used in the experiment.

Cytochrome *c* oxidase activity was determined by measuring cytochrome *c* oxidation as previously described [64].

### 4.6. SDS-PAGE and Western Blot

For immunoblotting, yeast cell extracts were resuspended at identical cell densities in sodium dodecyl sulphate (SDS) loading dye and lysed by boiling for 6 min and vortexing for 5 min with glass beads. Protein samples were separated into 7.5–10% SDS-PAGE gels and transferred to nitrocellulose membranes (Hybond-C, GE Healthcare).

The primary antibodies used were raised against yeast Tim22p (1:500, sc-14042, Santa Cruz Biotechnology, Dallas, TX, USA), yeast Cox2p (1:6000, 4B12A5, ThermoFisher Scientific, Waltham, MA, USA), yeast Cytochrome c (1:10,000, Davids Biotechnologie, Regensburg, Germany), yeast Pgk1p (1:30,000, 22C5D8, ThermoFisher Scientific, Waltham, MA, USA), HA (1:1000, Y-p11, Santa Cruz Biotechnology, Dallas, TX, USA) and *c*-Myc (1:1000, ThermoFisher Scientific, Waltham, MA, USA).

Secondary antibodies used were anti-goat IgG-HRP (1:5000), anti-mouse IgG-HRP (1:10,000, Molecular probes, Eugene, OR, USA) and anti-rabbit IgG-HRP (1:10,000, Sigma, St. Louis, MO, USA).

Membranes were incubated with WesternBright ECL (Advansta, San Jose, CA, USA), exposed to LucentBlue X-ray film (Advansta), scanned on a Molecular Imager GS900, and quantified using Image Lab Software version 6.1 (Bio-Rad, Hercules, CA, USA).

Full-length blots corresponding to the blots displayed in various figures and used for data quantification are provided in Figures S4–S6. 

### 4.7. Fluorescent Reporter Assay Measurements

Cells harbouring a GFP reporter for Rpn4p activity [33] were grown in YPGal until early-log phase. To assess Rpn4 activity under proteasomal stress conditions, wt cells were treated with 60 μM of MG132 (Merck, Darmstadt, Germany) for 2 h. Cells were then centrifuged, washed and resuspended in PBS buffer. Cells were acquired using the FL1 detector in a BD Accuri C6 Flow cytometer and data were analysed with FlowJo v10 software version.

### 4.8. β-Galactosidase Assay

Cells harbouring pRS415-AP-1-*CYC1*-*LacZ* plasmid [41] were grown in YPGal until mid-log phase. To assess Yap1p activity under oxidative stress conditions, wt cells were treated with 3 mM H_2_O_2_ (Merck, Darmstadt, Germany) for 1.5 h. The β-galactosidase activity was measured in a liquid assay using o-nitrophenyl-β-D-galactoside (ONPG; Merck, Darmstadt, Germany) as previously described [65] using 60 μg of total protein.

### 4.9. Oxidative Stress and DNA Damage Sensitivity

Wt, *cdh1*Δ, *yap1*Δ and *yap1*Δ*cdh1*Δ strains were grown overnight at 26 °C in YPGal medium until mid-log phase. Each culture was then diluted to OD_600nm_ = 0.1 and ten-fold dilutions were performed using PBS buffer. Cells were spotted in YPGal plates, used within 48h of preparation, supplemented with 0, 2.5 and 5 mM of H_2_O_2_ (Merck, Darmstadt, Germany) and 0.05% (*v/v*) of methyl methanesulfonate (MMS, ThermoFisher Scientific, Waltham, MA, USA). Cells were incubated for 2 days at 26 °C.

### 4.10. ROS Levels

Cells were grown overnight at 26 °C in YPGal medium until mid-log phase and incubated with 5 μg/ml dihydroethidium (DHE, Invitrogen, Waltham, MA, USA) for 30 min at room temperature in the dark. Cells were then centrifuged, washed and resuspended in PBS buffer. Cells were acquired using the FL3 detector in a BD Accuri C6 Flow cytometer (BD Biosciences, San Jose, CA, USA) sand data were analysed with FlowJo v10 software version.

## Data Availability

The mass spectrometry proteomics data have been deposited to the ProteomeXchange Consortium via the PRIDE [66] partner repository with the dataset identifier PXD039879.

## Supplementary Materials

The following supporting information can be downloaded at: https://www.mdpi.com/article/10.3390/ijms24044111/s1.
