# Supplementary material for: The APC/C Activator Cdh1p Plays a Role in Mitochondrial Metabolic Remodelling in Yeast
Source: Int J Mol Sci. 2023 Feb 18;24(4):4111. doi: 10.3390/ijms24044111 (PMC9967508; doi:10.3390/ijms24044111)
Supplement: Supplementary file 1 [file ijms-24-04111-s001.zip › ijms-2161178-supplementary.pdf]

SUPPLEMENTARY MATERIAL
Leite *et al*.

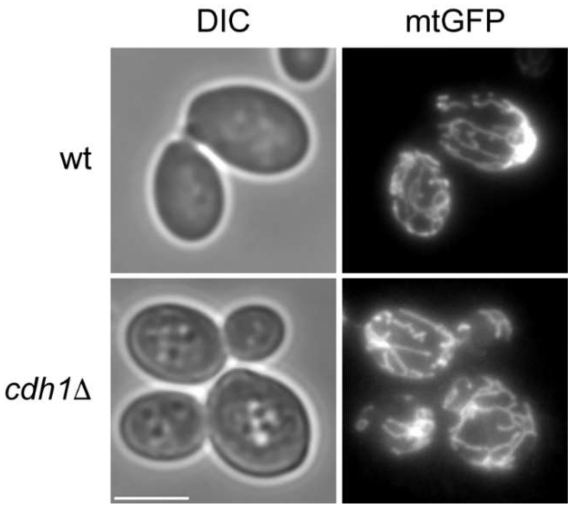

Figure S1 – Deletion of *CDH1* did not affect mitochondrial morphology. Yeast wt and *cdh1*Δ cells transformed with a pYX142mtGFP plasmid [Westermann and Neupert, 2000] were grown overnight at 26 °C in YPGal until mid-log phase. Cells were then imaged by fluorescence microscopy and images acquired by epifluorescence in a Zeiss Axio Imager Z1 microscope with Nomarski optics with an Axiocam MR3.0 camera and analysed using the Axivision 4.7 software. Images were collected at 0.4 µm z-intervals. Representative DIC images from 4 independent experiments are merged with maximum intensity projections from the mitochondrial GFP signal. Scale bar: 5 µm.

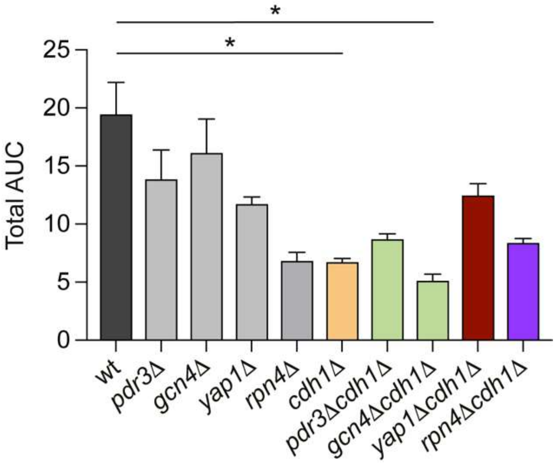

Figure S2 – Area under the growth curves calculated from data presented in Figure 3C, in arbitrary units (A.U.). Values presented are the mean ± SEM ($n = 3$), *, $p < 0.05$, Welch's t test of the area under the curve.

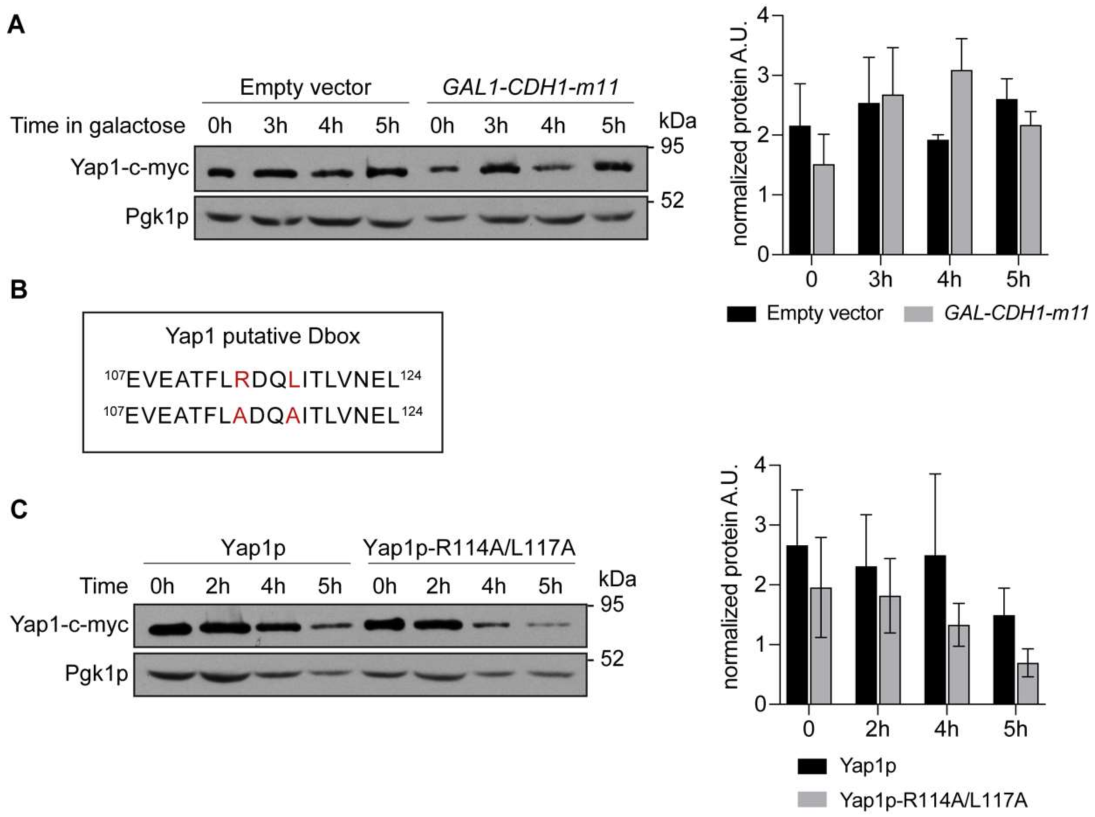

Figure S3 – Overexpressing Cdh1-m11 or mutating a putative Cdh1p recognition motif (destruction box) in Yap1p did not affect Yap1p stability. (A) *yap1*Δ cells harboring pRS315-Yap1-*c*-Myc plasmid and carrying vector for *GAL1* promotor-induced overexpression of Cdh1-m11 were grown in YPRaffinose. At early-log phase, 4% of galactose was added and cells were collected at indicated time points. Total protein extracts were separated by SDS-PAGE and analysed by immunoblotting using anti-*c*-myc and anti-Pgk1p (loading control) antibodies. A representative blot is shown. Graph represents the relative amount of Yap1-*c*-myc normalized to Pgk1p. Values are the mean ± SEM ($n = 2$). **(B)** Schematic representation of one putative destruction box (Dbox) in Yap1p predicted using GPS-ARM 1.0. **(C)** *yap1*Δ cells harboring pRS315-Yap1-*c*-Myc or pRS315-Yap1-R114A/L117A-*c*-Myc plasmid were grown in YPGal to early-log phase. The R114A/L117A mutation was introduced using the Q5 Site-Directed Mutagenesis Kit (New England Biolabs). Cells were collected at indicated time points and total protein extracts were separated by SDS-PAGE and analyzed by immunoblotting using anti-*c*-myc and anti-Pgk1p (loading control) antibodies. A representative blot is shown. Graph represents the relative amount of Yap1-*c*-myc normalized to Pgk1p. Values are the mean ± SEM ($n = 2$).

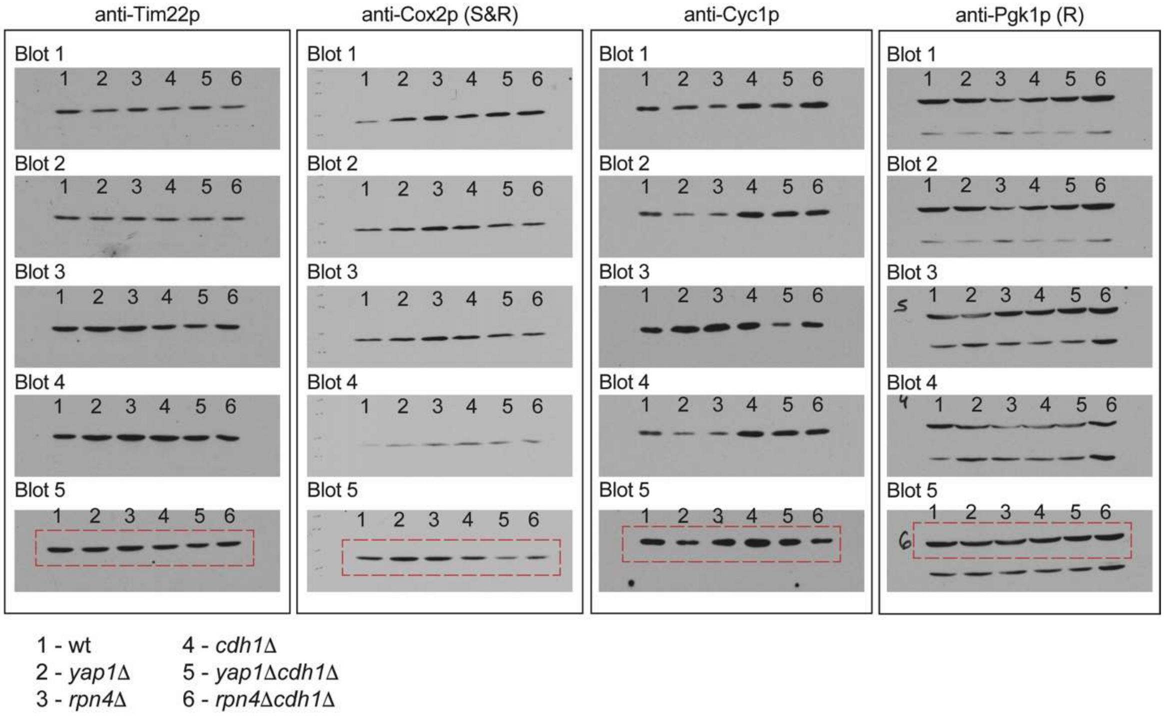

Figure S4 – Raw data of Western blot assays for bands displayed in Figure 4. Full-length blots used for data quantification with the indicated antibodies. Red rectangles are used to highlight where the bands were taken from. S&R indicates Stripping before Reprobing (R).

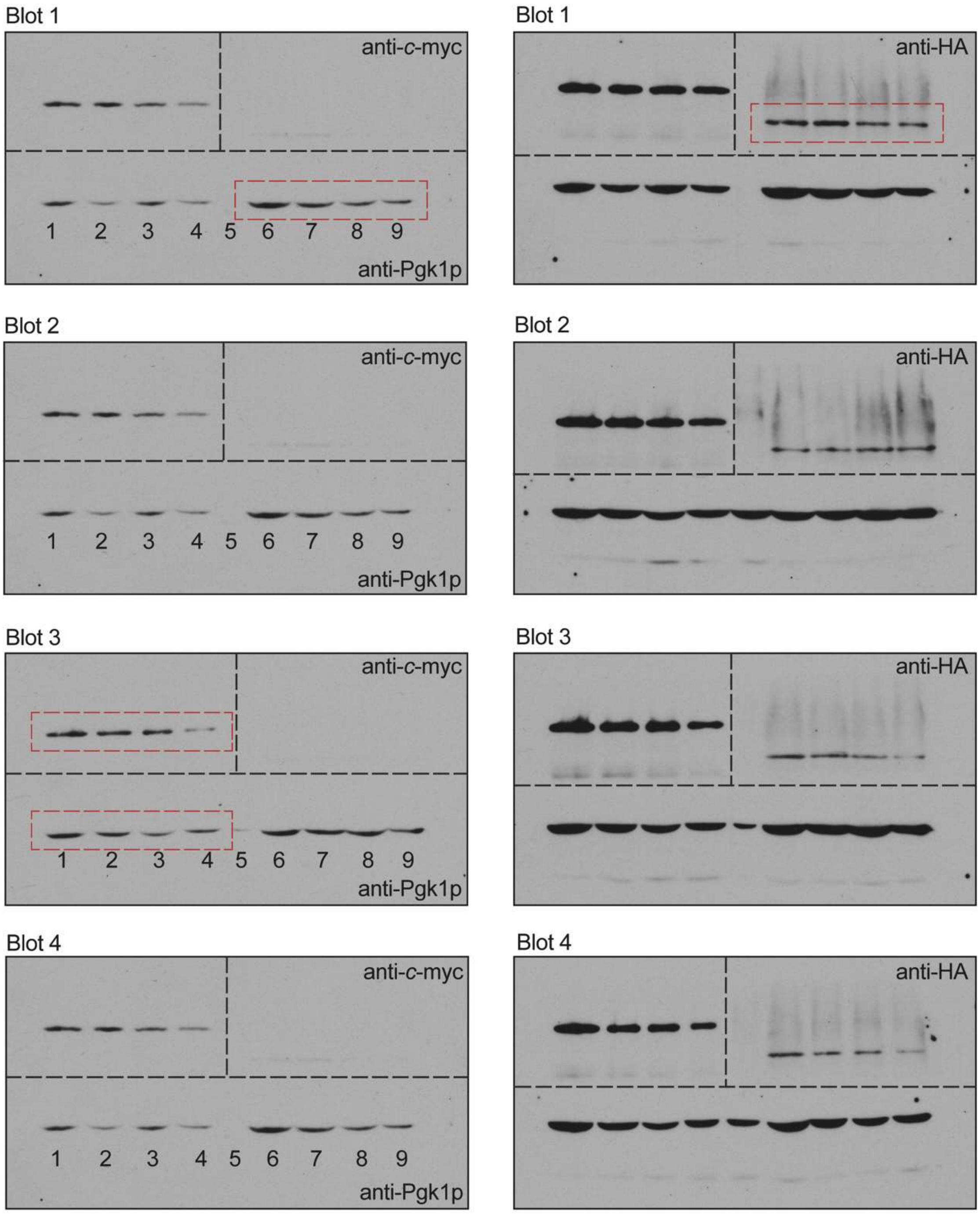

1 - *yap1*Δ + pRS315-Yap1-*c*-Myc OD 0.5
2 - *yap1*Δ*cdh1*Δ + pRS315-Yap1-*c*-Myc OD 0.5
3 - *yap1*Δ + pRS315-Yap1-*c*-Myc OD 1.0
4 - *yap1*Δ*cdh1*Δ + pRS315-Yap1-*c*-Myc OD 1.0
5 - no tag

6 - Rpn4-HA OD 0.5
7 - Rpn4-HA*cdh1*Δ OD 0.5
8 - Rpn4-HA OD 1.0
9 - Rpn4-HA*cdh1*Δ OD 1.0

Figure S5 – Raw data of Western blot assays for bands displayed in Figure 5 and Figure 6. Full-length blots used for data quantification with the indicated antibodies. Red rectangles are used to highlight where the bands were taken from. Dashed line indicates where the membranes were cut for antibody incubation and re-aligned for imaging.

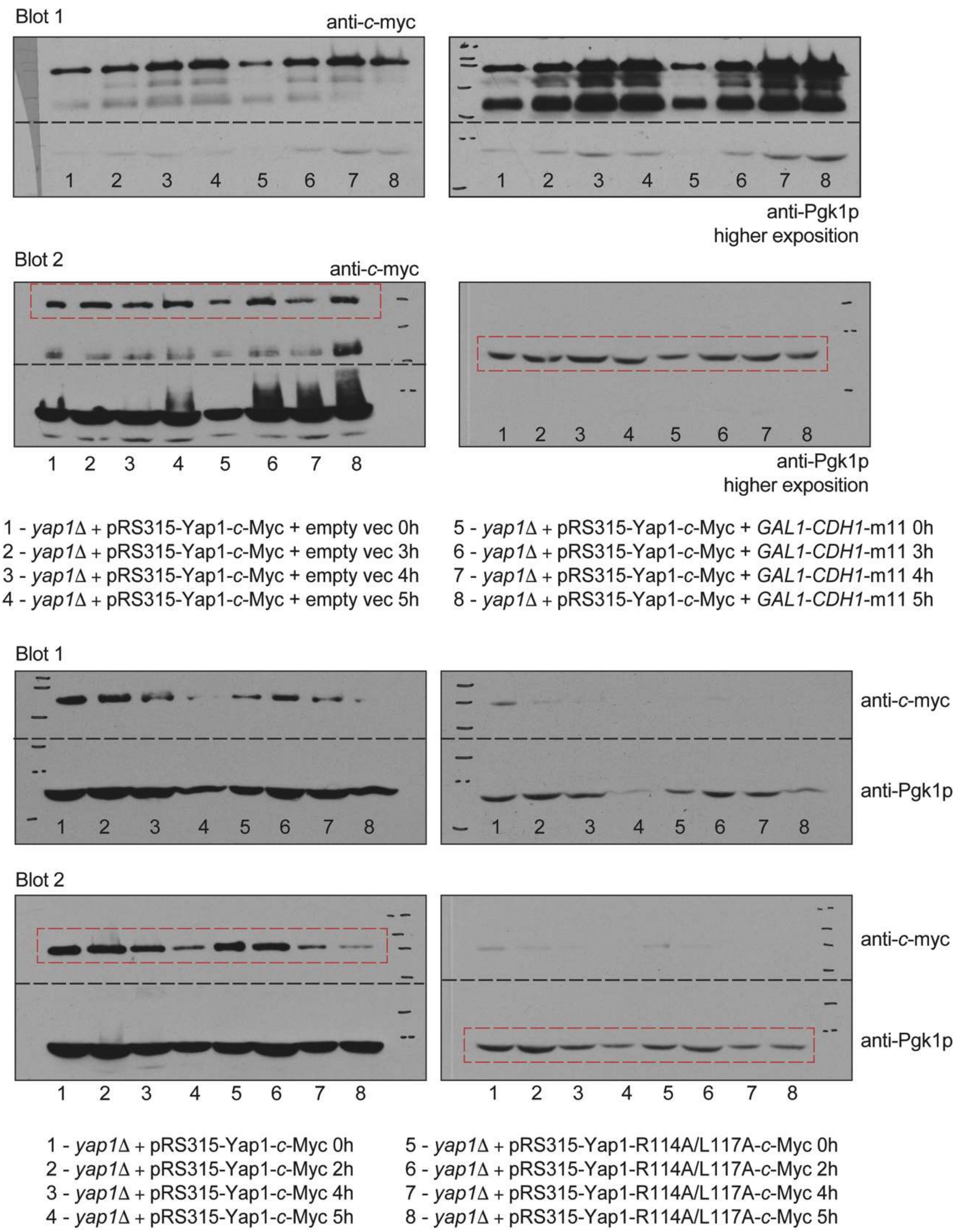

Figure S6 – Raw data of Western blot assays for bands displayed in Figure S3. Full-length blots used for data quantification with the indicated antibodies. Red rectangles are used to highlight where the bands were taken from. Dashed line indicates where the membranes were cut for antibody incubation and re-aligned for imaging.

Table S1 – *S. cerevisiae* strains used in this study.

Strain	Genotype	Source
BY4741 (WT)	Mat *a his3Δ1 leu2Δ0 met15Δ0 ura3Δ0*	Euroscarf
cdh1Δ::kan	BY4741; *cdh1Δ::KanMX4*	Euroscarf
cdh1Δ	BY4741; *cdh1Δ::HIS3MX6*	This study
yap1Δ	BY4741; yap*1Δ::KanMX4*	Euroscarf
rpn4Δ	BY4741; *rpn4Δ::KanMX4*	Euroscarf
gcn4Δ	BY4741; *gcn4Δ::KanMX4*	Euroscarf
pdr3Δ	BY4741; *pdr3Δ::KanMX4*	Euroscarf
yap1Δcdh1Δ	BY4741; yap*1Δ::KanMX4 cdh1Δ::HIS3MX6*	This study
rpn4Δcdh1Δ	BY4741; *rpn4Δ::KanMX4 cdh1Δ::HIS3MX6*	This study
gcn4Δcdh1Δ	BY4741; *gcn4Δ::KanMX4 cdh1Δ::HIS3MX6*	This study
pdr3Δcdh1Δ	BY4741; *pdr3Δ::KanMX4 cdh1Δ::HIS3MX6*	This study
rpn4Δ::hph	BY4741; *rpn4Δ::hphMX4*	This study
Rpn4-HA	BY4741; Rpn4-HA:HIS3	[Work *et al.*]
Rpn4-HA*cdh1Δ*::kan	BY4741; Rpn4-HA:HIS3 *cdh1Δ::KanMX4*	This study
